# Critical qualitative research on ‘madness’: knowledge making and activism among those designated ‘mad’

**DOI:** 10.12688/wellcomeopenres.16711.1

**Published:** 2021-05-06

**Authors:** Diana Rose

**Affiliations:** 1 Research School of Social Sciences and Department of Sociology, College of Arts and Sciences, Australian National University, Canberra, ACT2600, Australia

**Keywords:** critical theory; madness; mental illness; knowledge, power

## Abstract

This paper charts the background to a project which aimed to map the knowledge being generated across the world by people silenced for centuries – the ‘mad’: a term with derogatory historical resonances but which is now being reclaimed. The idea that those designated ‘mad’ can produce knowledge is novel: ‘mad’ people are imagined as lacking rationality, and incapable of producing knowledge; they are subject to epistemic injustice. Patient engagement in research has grown in the last 20 years but we lack methodological frameworks through which such knowledge can be surfaced. One goal of the project is to let the mad speak their knowledge, often practical knowledge. To do this we had to innovate methodology. Centrally, we refuse the distinction between theory and method for these are constantly intertwined in all research. Thus, what typically comes under ‘Method’ in background papers is infused with implicit conceptualisation. We carried out 48 interviews in North America, England, Australia, New Zealand, and Eastern and Western Europe. We argue all aspects of these interviews are radically different than is usual for exploratory research in this area. Psychiatry is not central here – it is present only when present in the words of our participants; situated in material and symbolic spaces. We also seek to move away from the individualising therapies of medicines and psychological treatment because they strip participants from their situated realities. Psychiatry enters also because of what it does *not do* – engage with the life world of its patients. We call then for ‘recontextualisation’ of madness at all levels. The project was user-led and all researchers had experienced distress and responses to it. Future papers will develop and demonstrate this approach.

## An initial clarification about the scope of this paper

This paper gives the background to a study aiming to surface the knowledges being generated by users of mental health services, survivors, refusers and people with psychosocial disabilities. In other words, people deemed deficient in their capacity for knowledge making. The proposal for the project stated that it would be ‘global’ in scope. This turned out to be an impossible task because of team dynamics, and particularly due to differences with regard to the Global South, where positions on racialisation became entrenched in a very strong way. This has had the consequence that a single background paper became unfeasible. This paper develops the framings and arguments which are more germane to the Global North, but including people with intersecting positionalities. I have not here discussed issues pertaining specifically to the ‘Global South’. While other members of the team contributed to the thinking behind this paper, the synthesis which this paper represents is mine and I take full responsibility for any errors. The point of this short paragraph is to be transparent about the circumstances surrounding this work Full details of the researchers involved in the EURIKHA project can be found at www.eurikha.com.

## Introduction

Why don’t those involved with mental health research policy, researchers and members of the public themselves listen to the voice of those with ‘lived experience’, to use a topical term? Many state that they are doing this, and multi-authored research papers increasingly claim to include people with ‘lived experience’ among the authors ( Holmes *et al*., 2020). The recent *Lancet Commission on Global Mental Health* argues that “engagement of civil society with mental health should be increased, in particular of people with lived experience of mental disorders’ and a recent Editorial in *Lancet Psychiatry* acknowledges that “global mental health” contains assumptions about knowledge and expertise that are inimical to the very populations it purportedly sets out to help ( Patel *et al*., 2018). Fundamentally, we must recognise that expertise comes from individuals and communities in specific social, cultural, and economic environments, rather than the world being a blank slate on which supposedly value-neutral, technocratic solutions can be imposed.” So, there have been steps forward and they are important. But in this paper I argue that they are radically insufficient because of often tacit assumptions about lack of capacity of those with mental health challenges and denial of structural inequalities. The insufficiency is with the experts this time but is deeply embedded so difficult to recognise. It revolves around the power of omission and includes not addressing the challenges which would facilitate a real voice for service users, nor the challenges which would which face survivors in making possible a real shift in how ‘mad’ people are imagined and treated. Nor do said ‘experts’ recognise themselves and their institutions as barriers to such changes. This includes their own inability to listen to the voices of experience and so prevents the inclusion of persons with mental distress in these debates. ‘Mad’ is a term with derogatory historical resonances but which is now being reclaimed by those critical of the idea of madness as some kind of deficit of reason. We must recognise the many hurdles faced both in generating new knowledge and establishing how it can be used in mental health specifically to ground new developments. I therefore here set out an alternative, based on the developments in theory and method utilised in a project which aimed to explore the ways in which people designated ‘mad’, specifically the mentally ill, survivors and people with psychosocial disabilities, are developing new forms of knowledge about their distress, the forms of support associated with that knowledge and wider questions concerning their relations with ‘mainstream’ academic research. This alternative partly lies outside the mainstream altogether and so it is not just to ‘listen’ but with the purpose of ‘change’ – radically

## Terminology

I started this paper with the term ‘lived experience’ because it is currently topical. But it should be noted that ‘mad’ people who are changing knowledge and practice have in the past and still now use other terms. The unqualified term ‘experience’ is significant too and I shall return to it. The variety of terms is not random and they can indicate the groups’ activities, thinking, and practical positions. For example the word ‘consumer’ was used for many years in Australia whilst an activist group in the UK chose ‘Survivors Speak Out.’ This indicates a wide difference in vision and political orientation from just these two and there are many more. This phenomenon has been recounted and analysed in detail ( Lyon & Mortimer-Jones, 2020). I will use ‘lived experience’ where relevant but invoke other terms if they are used in work we are citing.

The project to be discussed is called EURIKHA – Explorations in User Research, Impact, Knowledge and Activism – and all the researchers in the project team were, or had been, users of mental health services. As a group of researchers with experience of mental distress and responses to it, including those who have experienced what are commonly referred to as severe mental disorders, we were well-placed to undertake this work. What this paper aims to do is articulate some of the fundamental elements that have arisen, changed, developed, split and were revisited in the time line of the project. The research focused on the last few decades, starting in the early 70s when the modern ‘user and survivor movement’ in mental health developed ( Campbell, 1985; Davar, 2013). We aimed, in addition, to compare the knowledge produced by service users to mainstream knowledge about ‘mental illness’.

Of course, we recognised that neither mental health service users or ‘mainstream researchers’ are homogeneous. Those designated ‘mad’ are not a uniform group either and attention to difference is paramount if we are to understand the complexities at stake, including the hierarchies and expertise *within* the field as well as its relation to others. The concept of intersectionality can help here, although applying it to mental health is not straightforward ( Fricker, 2007; Kurs & Grinshpoon, 2018). This ‘mainstream’ – that is to say the body of accepted knowledge and those who create and legitimate- is also not homogeneous. What is taken to be knowledge varies over time as does the status accorded it. It also varies across place, and in our approach, the local is as important as the general. Thus we aligned ourselves with Haraway’s ‘situated knowledge’ but paid more attention than she does to the material aspects of a setting ( Haraway, 1988; Nazarea, 1999). Some features of the mainstream knowledge at play here are well known. They are persons or groups with the authority to speak and write *about* us because it is assumed we cannot speak for ourselves. We argued that knowledge of the worlds of mental health and associated arenas will always be incomplete if undertaken from these mainstream perspectives. Indeed, this may be deeply flawed ( Double, 2002). We aimed to find out about the kinds of knowledge people designated ‘mad’ are generating, their form and their content, how they are situated and what the symbolic and material requirements are for such knowledge to grow and act as an antidote to the constant ‘othering’ to which we are subjected. We also focused on the aspects of life that are *missing* from mainstream knowledges. Silence speaks volumes.

The paper focuses on 48 interviews from survivors in the Global North, but as will be seen these interviews are treated differently from the way interviews are usually positioned in a project. Allied to this, we did not systematically review the literature. We conducted a rapid review but this is unsatisfactory when much of the literature is not peer reviewed. Therefore we positioned the literature as part of the ‘context’ – immediate to an interviewee or more widely. We were attentive to things that our some participants had published, both to give us a greater sense of the contexts from which they were speaking, and also because in their interviews, they sometimes expanded, and sometimes contradicted what was in that written material. And of course some interviewees had a public profile but others did not, and were much more focused on local activism. For example, one participant talked at length about having an intersectional identity and the relation of this to activism, but their published writings are quite conventional. By contrast, another participant with a public profile spent most of the interview discussing their ambivalence about being identified as a service user. Therefore we did not confine ourselves to the peer reviewed literature and go beyond even the ‘grey’ literature and other forms of representation ( Sexton & Sen, 2018). The rationale for this should become clear.

This paper is not a research protocol. Our research is complete. Because it was an exploratory project, we could not lay down in advance the ways our approach would develop, even though some aspects grew out of work that we had used previously but not systematically. I would like this paper not just to explain why mad people are imagined as they are, but also to change dominant narratives and associated practices and to draw upon our own engagement with mental distress, variations in the ways people live and think, and the novel debates that characterise the field. I see this as a ground clearing exercise that is necessary for subsequent papers which will lay out more substantively the lineaments of this field of survivor knowledge in the early 21 ^st^ Century. So, this paper is not value-neutral but seeks to demonstrate the generative nature of knowledge making by people who may now be recognised as able to provide anecdotes and stories of their own lives, but are not believed to be capable of generating knowledge. And as for stories more generally, only certain ones will do – mostly ones of recovery made possible by a psychiatrist or medication ( Costa *et al*., 2012; Slade, 2009). This has been called ‘disability porn’.

## Theory and methods

Introductory papers such as this often have a section called ‘Theory’ and another section named ‘Methods’, implying that these are at least partially distinct in a body of work. I have not divided the paper in this way because one of our central arguments, and the one to be articulated here, was that the conceptual and methodological work are intertwined throughout. Consequently, I will treat them and their interrelations together. Existing literature on ‘concepts and methods’ is almost exclusively statistical and gives primacy to data rather than conceptualisation in formulating conclusions ( Gentle *et al*., 2012). There is a large body of literature on the development of measurement and scales in relation to mental health, but where this does discuss theory and method the its main focus is again statistical, in the form of psychometrics. Mental health ‘needs assessments’ often use this approach ( Rasch, 1961) as do tests of ‘intelligence’, ‘achievement motivation’ and so on ( Michell, 1997). Such work claims to be ‘hypothesis-free’ and that the results arise from the data itself. We do not accept this view. For example, in epidemiology the categories through which information is collected puts strong constraints on what the data can tell us. An example is contemporary surveys which use socioeconomic categories in an economy that is first, primarily a knowledge economy and second, a system invented before economies characterised by austerity and zero-hour contracts came into being. Similarly, psychiatric epidemiology uses formal diagnostic categories and researchers here seem oblivious to their power. Categories such as schizophrenia or major depression function as if they were ‘natural kinds’ and thus go unquestioned ( Kohrt *et al*., 2014; Young, 2004), see Hacking ( Hacking, 2007). Even in work that calls itself ‘interdisciplinary’ the focus of discussion is the *difficulty* of working in an interdisciplinary way, not arguments that would facilitate it. The whole architecture of scholarly work as constituted by ‘disciplines’ is taken as a given and to disturb this is unthinkable for most ( Sewell, 1989). Of course, there is much critique of psychiatry, including of its categorical diagnostic structure ( Haslam, 2002). But our task was to surface the positive knowledge of survivors. Some takes the form of a critique of psychiatry, while for others, psychiatry is not relevant or is to be avoided. Our project centred on the survivor perspective and therefore psychiatry itself, although exercising power at multiple levels, will be discussed only when the data or preliminary analysis demand it. Psychiatry was not our starting point; survivor perspectives are.

### The role of speaking back

Historically, the ‘mad’ have been written and spoken *about* but until recently there has been no voice for this group. This is because persons designated ‘mad’ have been portrayed as unable to think, as lacking reason, as incapable of forming relationships: madness is an individual aberration. The ‘mad scientist’ is not a joke: it is a paradox. Experts such as psychiatrists and all the ‘little psys’, educationalists, priests and therapists have been thought to have a monopoly on what madness is, and what should be the appropriate professional and public response. People with psychic challenges are set apart from the ‘normal’ and can be given treatment deemed curative by professionals, even when it is often experienced very negatively by survivors. This includes treatment they do not want or which harms them in many ways. We, living under these descriptions, have no voice of our own because it is has been assumed that any knowledge we create will be incoherent, lacking reason, detached from reality and severed from social significance by virtue of these attributes ( Foucault, 1967; Porter, 1987; Porter, 1988). It is still the case that ‘mad’ people are held not to be able to give an account of their own experience, and only rarely have ‘experts’ tried to find meaning in their own explanations of their predicament ( Kohrt *et al*., 2014) Jaspers’ General Psychopathology is a good example ( Spitzer, 1988). Fricker’s concept of ‘epistemic injustice’ is useful for our analysis ( Fricker, 2007). Epistemic injustice is to define a person as not a credible source of knowledge. Fricker’s original analysis was concerned with women and feminism. But Foucault made a similar point 50 years ago specifically about ‘mental illness’. He writes:

          “The constitution of madness as a mental illness *thrusts into oblivion all those stammered imperfect words without fixed syntax* in which the exchange between madness and reason was made. The language of psychiatry, which is a monologue of reason about madness, has been established only on the basis of that silence” ( Foucault, 1967) , emphasis by author)

Foucault is being quite specific here. It is psychiatry that has constituted madness as a mental illness, its practice renders the ‘mad’ person the antithesis of reason, and on that basis the speech of the ‘mad’ person is not even heard, or heard only as a symptom of their madness. It is this last part of the quotation that is the most important, and often overlooked. The book – Foucault’s *Madness and Civilisation* – has had many critics, for romanticizing madness whilst at the same time being not just Eurocentric but specifically French in content ( Porter, 1990). But it is a place to start, a point of entry but with fluid and provisional boundaries and open to revision. A place to start but not an origin. Its significance lies in approaching madness as a space where specific knowledges break out only to be silenced, rendered illegible, invisible, for three centuries and more. The task is to trouble that silence and let madness speak.

### Our project specifically

We will now see how this general background relates to our specific project. Although the researchers were from different disciplines, most had in one way or another or at some point adopted the style of papers in mainstream psychiatry and psychology. These disciplines take the individual as their unit of analysis and hence there was a tendency to see what we were looking at as the property of individuals. This was a limitation which we pushed against constantly. Might it have been enriched by drawing on other disciplines? The problem is that anthropology, the humanities, social science and neurology may represent things differently, but they share central themes: madness as: bestial, possessed, dangerous and unpredictable. Since the Western enlightenment the ‘mad’ have been imagined as lacking reason and unable to engage in the structured activities required for society to function, such as work in the open market. We are touching a central tenet of critical theory – the valorisation and articulation of knowledge generated by marginalised groups. 

Critical theory was first used as a term by the Frankfurt School, a group of East Germans of Marxist persuasion whose fundamental goal was to show the power wielded by cultural hegemony instead of relegating it to the ‘superstructure ( Forchtner, 2011). The Frankfurt School concerned itself with the overall form of oppressive cultural structures and focused its analysis on social class ( Geuss, 1981). There are many strands to this school of thought that can be useful. But major problems arose when diverse subjugated social groups found Marxism inadequate for understanding their specific material and symbolic situations. Instead of relying on Marxism, new theories of knowledge emerged from these new collective forms of action and resistance, and the knowledge makers were also activists - feminist, post-colonial, LGBT+ groups and more. Significant for our work was the development of critical theories and practice in the disability field. So, against the background of the Frankfurt school, we understood critical theory as knowledge produced by marginalised groups to understand, develop and change their situation. Authors in many different fields have taken this approach; feminist studies ( Harding, 2008); post-colonial work ( Danius *et al*., 1993); queer theory ( Butler, 2011), disability studies ( Oliver, 2013) and work on intersectionality ( Bell, 2010; West, 2020). The evolution of our concepts of ‘knowledge’ was built on some of the elements of this work. It changed over time but the trajectory of these concepts in EURIKHA is important. The original project proposal put a heavy emphasis on ‘user-led research’. In a very short while we realised that this was a narrow and elite view of knowledge, both in terms of the settings where knowledge developed and in terms of ontology. Knowledge arises in the academy, it implied, and this is its natural home. Natural and prized. But most of the researchers in EURIKHA had been activists in some manner in the mental health field for many years before this project was proposed. We knew that activist and self-help groups had produced analyses of experiences that reconfigured and overturned venerated psychiatric diagnosis: eating distress and self-harm ( Pembroke, 1994; Pembroke, 2005); hearing voices ( Blackman, 2001; Corstens *et al*., 2014); bipolar disorder ( Martin, 2009); PTSD ( Kaplow *et al*., 2006). Most of these authors had first-hand knowledge of the descriptors they had been given. The work was innovative: its reception by mainstream psychiatry was predictable – it was usually ignored. But for us, campaigning and self-help organisations were legitimate sites of knowledge production just as much as the academy. Another setting was peer support ( Faulkner & Kalathil, 2012; Voronka, 2017). The realisation that you were not alone with your distress and that reflecting and acting together produces a different set of understandings and different subjectivities was a key moment for some. And research can be political. Indeed, the idea that most research is value-neutral is something we rejected. As our concepts of knowledge deepened and widened, we did not abandon the focus on user-led research; rather we tried to revalorise knowledge production by widening the settings included and focusing on a world beyond that which had attributed a pathology to an individual.

### Hierarchies of knowledge

There is a hierarchy of evidence *within* the academy but also a hierarchy between academic knowledge and the rest. Diversifying the sites where knowledge was produced was central to the conduct of the project. It had always been agreed that we did not want to interview a random or even representative group of participants. The people we wished to engage in our work were people and organisations who were producing new knowledge. It did not need to be high theoretical knowledge, it could be and often was practical knowledge ( Telford & Faulkner, 2004). And so we set about a ‘mapping’ exercise where potential participants and their organisations were entered on a spread sheet with the single eligibility criterion being that they were actively pursuing alternative analysis of their own and others’ conditions of existence. We began with people we knew or who were well known, but through a process of snowballing the map grew until it had more than 300 names and organisations.

People with mental illness are seen as cognitively and emotionally compromised and are usually treated with medication or some form of psychological intervention. That is, they are treated conceptually and treated in practice as individuals with no social context, no social determinants that could account for their dilemmas, no focus on poverty or familial care or constraint, no focus on norms and the consequence of breaching them. Such individuating inhibits collective organising and the development of campaigning goals and understandings. This tendency to individualise is growing within societies characterised by austerity today, leaving people to subsist on few resources and with few friends ( Friedli & Stearn, 2015; Lasch, 1980). Some knowledge makers in the arena of mental health are in hiding and this had important implications for their participation in this project.

This account makes the process seem one of linearity as if we were proceeding step by step. This is inaccurate as the ‘steps’ overlapped and influenced each other. Participants in the project were encouraged to revisit parts of their interview rather than give a single narration, at the time of the interview itself or later through comments.

It should be noted that the idea of ‘context’ is complex. The term is central to the fields of physics and engineering but is also of great significance in anthropology where ‘context’ functions at many levels including the interpretation of their characteristics and situations by people themselves ( Dilley, 2002). We also have the idea of C-M-O (Context-Mechanism-Outcome), coming from realist evaluation ( De Souza, 2013). These three approaches, using the same word, could not be further apart. In the critical realism approach, ‘context’ functions as a black box – taken for granted and never unpacked ( Næss, 2004). Much science and technology studies (STS) concerns itself with physical science and information technology. But there are exceptions, for example, the work of Callon and Rabeharisoa with disabled people and marginalised groups ( Callon & Rabeharisoa, 2003; Callon, 2009). But the basic point – that ‘context’ remains both central and not articulated – holds in social science too. And, it will be argued that it can resonate with the field of ‘psy’ research and practice where madness is seen as a phenomenon with no discernible context other than demographic sheets sometimes filled in by participants, in research or clinically. These are treated as independent variables in any analysis which is usually quantitative. So ‘context’ can mean many things and later I will describe how Western psychiatry treats the individual as though they had no material or social elements to their lives – everything is focused on a diagnosis or a treatment. It is true that there are many ethnographies that touch on the experience of madness, and these are rich in 'context'. But, in common with so many fields, all these arguments are almost always made by academic or other ‘experts’. None of them include service users in the author list, and references to arguments where service users could be central to the making of new knowledge and strategies for change are absent. 

### Ethics

Our approach was difficult for some to understand. For example, we applied for ethical approval, and were indeed granted such approval: (REMAS) LRS 16/17 4502. Despite this, we had to revise and resubmit to counter some misunderstandings. For example, we suggested a simple question “What triggered your interest in this field?’ The response of a member of the ethics committee was to ask whether there would be a psychiatrist available. In psychiatry the word ‘trigger’ is used specifically as a cause of an episode of mental illness. The committee, reading a submission on mental health, had assumed that we were asking if the person felt ‘triggered’ by the interview setting. We had to submit a revised application to explain this and similar misunderstandings. It is hard to challenge conventions, especially in the area of mental health where expertise is thought to lie with psychiatrists by many people, especially academically

### Participants: who did we talk to?

Before proceeding with our argument, I can anticipate criticisms of the order that our work was not ‘rigorous’ or not ‘real research’ because it has a biased sample. This kind of response is nothing new: in a field that has established itself with strict rules about what counts as knowledge, introducing new ideas, and especially those emanating from a subjugated position are likely to be suppressed, ignored or co-opted. Scholars in the field of science and technology studies have given us many detailed examples of how and why some new knowledge is suppressed whereas others will grow. In entitling his book on science *Never Pure*, Shapin demonstrates that sociability, power and value underpins much of science, and not veridicality as such ( Shapin & Fuller, 2015). Research and scholarship are shaped and set by social relations and social structures. This does not imply that knowledge that survives is ‘better’ as Shapin makes clear. It can be quite the reverse and so an adequate theory of the production of valid knowledge would not be confined to issues of logic and syllogisms , or even reliability or validity – we need to take account of values throughout and much more besides ( Ajei, 2007). Our approach, seeking explicitly to bring forward subjugated voices, necessitated us rejecting conventional ideas of sampling and representativeness, which are themselves formed within particular disciplinary structures.

The above discussion of sites and groups as settings for knowledge generation goes hand in hand with the development of the ‘mapping’ exercise described above. Names and organisations of potential participants were entered into an Excel spreadsheet together with some of the other relevant background information. As our conceptualisation of ‘knowledge’ developed so did the kinds of people we wanted to talk with and where. We made every endeavour to find out which organisation(s), if any, potential participants belonged to because of our widening concepts of knowledge and our misgivings about individuals being the sole source of knowledge, especially that which chaffs the mainstream. This is not a novel observation: many have written about the foregrounding of the individual as knowledge producer in modern Western societies ( Triandis, 2018). As I have said, theoretically and empirically, both psychiatry and psychology work with individuals in an almost context-free way and as such shape subjectivity so that the person becomes a ‘case’ ( Flore *et al*., 2019) We therefore sought out participants whose perspectives were explicitly socially grounded. 

Marginalised groups are hard to contact. We tried hard to find people who were not high-profile – that is not high profile in a group already marginalised. Our success here was partial and had we had more resources the stories and persons involved would have had more detail and depth. But had we tried to find a ‘representative sample’ we would have amassed much less detail about the diversity of knowledge that was being produced, or at least painted a different picture. Our method of choosing participants violates every rule in the book about sampling but it was consistent with our primary goal – to understand knowledge making by people and groups who were designated mentally compromised. Those very people who are envisioned as not able to produce knowledge at all.

### Interviews and context

In keeping with our decentring of the individual, we found out as much as we could about the lives of the persons on the map, their workplaces and their identifications. We attempted to ‘recontextualise’ the person by placing them relative to their own and group history, the work they did whether informal or formal, their familial situation, cultural norms, the nature and number of links with other marginalised groups and how each person saw themselves in the current moment of change, both individually and collectively. Marginalised groups are difficult to identify. In keeping with our evolving concepts of knowledge, we tried to find people in grassroots organisations as well as prominent individuals. This was an immense effort and we placed no bars on where to find information about an individual or group as long as ethical principles were followed. Sometimes an informal conversation took place before the person ‘went on the record’. This differs from much qualitative research where the method of data collection is standardised, and, for example, projects specify the number of people to be interviewed each week. Commonly this is graphed – indicating the number aimed for and number actually interviewed, with the discrepancy between the two a marker of success or failure.

The prized ‘outputs’ in the epistemic West are peer reviewed papers. There is a growing literature on ‘user-led research’ but, as a consequence of our developing concept of knowledge, we did not privilege the peer reviewed literature – we also went far beyond the ‘grey literature’. But many people, due to their subjugation, do not have a public profile at all. They may be important advocates and activists locally, but their work is not visible outside this in any detail. This led to a biased participant group but in a way that the term ‘bias’ is not commonly used. Those who were wholly or partially associated with academia were the easiest to find and to find out about, which means that this form of representing knowledge has a head start. This was an ongoing struggle in the as the initial framing of the project did not give enough weight or resources to groups for whom academia is totally irrelevant. We attempted to correct this as we proceeded but we were not entirely successful.

There is another critical point that should be addressed. Some of the people we approached to be interviewed simply refused. In the main this was a matter of trust and power. The project was situated in the leading psychiatric research and teaching institution in Europe. This was our institutional context and it was a barrier to speaking with some of the people we were anxious to interview. For them, quite understandably, we were asking for their views from the vantage point of academic elitism and salaried Western academics. One important reason seems to be that they did not trust us to understand what they had to say, that we would warp it or turn them into mere data points. So, our own institutional position constrained the information we accessed.

In sum, try as we did, our positionality meant that certain key groups of people were not represented to the degree that should have been. Either they were the most silenced group, or they were cynical about our motives or both. On the other hand, the work surrounding each interview sets this project apart from most qualitative research where at most there is a demographic form for each individual to complete. Of course, this is in some ways different from how other disciplines and non-academics proceed, but it is the hegemonic orientation ( Woods, 2011).

In formal terms, it is important to note that interviewed face-to-face gave written consent and completed a consent sheet. Those interviewed by Skype gave verbal consent, and this was properly noted and filed.

### Conducting the interviews

In our first discussions about the interviews we thought they would be amenable to an oral history approach, as it was developed in the 1970s and as in the more complex form that it now takes. A full account of oral history and its strengths and weaknesses would be another paper. But fundamentally, the goal is that the speaker has control of their narrative for they are ‘telling their story’ ( Portelli, 2009). Here there is an apparent levelling of power in research, but there is controversy about this ( Molden, 2016). The power relations in an oral history interview have been much discussed, especially by feminist historians, which has often been linked to the question of whether it is possible to ‘bracket out’ the researcher ( Scanlon, 1993). These are important considerations, and they stand, but there is a further issue about a person simply telling their life story, if the interview seeks to capture specific moments or perspectives that are important for the project as a whole. Exploring the knowledge potential of service users’ lives and organisations thus needed to incorporate some key questions on our part. Our initial idea of using oral history had to be modified because, as we worked on the topic guide (see below) we realised that there were certain key issues that often needed prompting if we were to conduct our work so as to explore questions of marginalisation and subjugation. These themselves are not just ‘descriptions’ of events or feelings - they often become evident only if viewed from a concern with inequality and social justice. A good example again is ‘epistemic injustice’, a concept alluded to earlier to show how some groups of people are marginalised at an epistemic level – they cannot think or speak either about themselves or others and so are not credible knowers. As we describe below, we coded the interviews, and ‘epistemic injustice’ was a sub-code of ‘theorising’ in the coding frame. However, it turned out to be by far the most often used of all the sub-codes under that ‘master code’. This was not just a question of counting the number of appearances of a code, and of course people didn’t have to use the term itself: identifying epistemic injustice was an act of interpretation. So our approach was not merely one in which the coding frame drove the analysis: the interviews themselves were constantly changing the concepts used in the analysis, and its focus.

We therefore identified some questions that would make our concerns transparent. How best to hear about issues of power and knowledge, history and theorising whilst at the same time trying to observe the principle that the relation between researcher and participant should aspire to one of different expertise – different but of equal and complementary value. The complexities here were again prompted by feminist oral history research where one much discussed issue was whether women interviewing women would elicit richer and deeper information from people and groups who often did not have a voice ( Turnbull, 2000). These debates have been raised in user-led research, asking whether having someone with a history of using mental health services (or avoiding this) makes a difference to the interview ( Newman & Clarke, 2018). It is suggested that if this did not fully cultivate a space of equity, it would broaden the frame of what is actually sayable ( Barnes & Wistow, 1994). A similar debate exists in relation to racialised groups and oral histories of people of black and minority ethnicities ( Kim, 2008).

Thus, to put the process in formal terms, interviews were guided by a topic guide, but in some cases, as normal, were tailored to the specifics of the interviewee which we had discovered during our mapping work. Interviews were all performed by members of the team, except for two (in Chile and Japan) where local interviewers were used, and translations undertaken. All interviewers were trained on the interview protocols.

### Coding

Debate about specific aspects of research, and especially technical ones, runs the risk of abstraction from the approach as a whole. To consider ‘interviews’ as an isolated method loses its connection to the lifeworld of the speaker and the overall methodology. It was finally decided to blend the principles of oral history, its attention to the research relationship and power dynamics in the interview setting with a flexible qualitative methodology. This methodology entailed analysing the text of the interviews with a coding frame to assess the use of words, concepts and arguments in the accounts given by our interviewees. We built a ‘coding frame’ which foregrounded seven major categories and each had sub-categories with some ‘codes’ free floating. We used NVivo to code the transcripts and made use of the ‘annotations’ feature available. This usefully allowed us to comment, abstract and compare, both within and between transcripts. This was one way of developing theory and the use of annotations increased as we became familiar with the deep content of the transcripts. We were also able to see which codes had few or no references. That in itself was a finding and did not mean the concept was unnecessary. Rather it could challenge us so that we searched for meanings where there was a reason they might be absent. For example, when we considered one interview where very few different codes were used, it became clear that this was because the participant was completely focused on the question of what it meant to be a survivor researcher and activist. This revealed the contrast with other participants who cast this question of subjectivity intersectionally, exploring their roles in different identity groups rather than dwelling on one.

Germane to the work on the interviewer/participant relations was that all the interviewers were service users. The ‘evidence’ regarding appropriate interviewers is sparse and lacks discussion of what was of most significance to us – that it should be connected up with other aspects of the project and thus elicit perspectives on our key questions which would not surface had the interviewer not been a service user. An example would be the ability to elicit criticisms of other strands in the user movement which some people avoided in case it was seen as a ‘betrayal’. In other instances what was said by our participants was strategic in the sense of Spivak’s concept of ‘strategic essentialism’; those times when diversity is glossed over to pursue a particular goal ( Danius *et al*., 1993; Spivak, 1988). The ‘method’, including the status of the interviewer was therefore consistent with and partook of the theoretical arguments set out above.

### Instruments

The initial project specified three groups of participants: historical figures, those from the Global North and those from the Global South. These groups were not distinct, many people could be positioned in more than one. We formulated a rough interview guide for those from the Global North and piloted it. This was aligned with the project’s initial aims but crafted to avoid favouring material on user research. The guide was indeed a ‘guide’ and this was made clear at the start. And, of course, the distinction between Global North and South has been widely critiqued ( Müller, 2020)

The aim was to have the topic guides for the three groups ‘mirror’ each other but this proved unfeasible in terms of how people talked. They overlapped of course, but the pilot coding showed different patterns of discourse. It also showed that relations between a participant’s personal history and the history of collectives they belonged to was quite complex, raising questions about what ‘diversity’ meant and also the nature of the relation between an individual’s history and that of the collective. So some participants spoke almost exclusively about collectives in the past, whereas others recounted their current concerns with user-led research ( Weedon & Jordan, 2012). We found stories of successes, failures and nostalgia in different transcripts and sometimes within the same ones. I doubt whether our participants would have been so forthcoming had the interviewer not been a service user. A second topic guide was formulated to be closer to the dominant concerns in other regions and settings, especially the Global South. In practice, background work showed that a participant had very distinctive experiences and reflections and here the ‘topic guide’ was quite malleable and tailored to that person.

The interviews were either conducted face to face or via Skype using the audio software, Audacity, to record the interview. The fact that English was not the first language for some participants added another layer of complexity. Within the Global North, we used interpreters three times and this was not without problems ( Ingvarsdotter *et al*., 2012). In Australia and New Zealand we conducted five interviews with indigenous people and the comparison with the interviews with people of predominantly white ethnic groups was revealing, particularly in relation to stigma: one participant told us that ‘mental illness’ was stigmatised by their community, not for itself, but because it was Eurocentric and alien concept ( Smith, 2013). Another difference appeared when a Maori participant criticised the very idea of a ‘meeting’ because of the differences between Maori practices and the mainstream. They were angered to be confined to an agenda, rules about turn taking, the ‘meeting’ ending ‘on time’ regardless of what was left to say and, crucially, the absence of food.

Transcription was checked first by the researcher who conducted the interview. Then, in order that participants could hear first-hand how they were coming across, each was provided with a copy of their interview to check that it was faithful. Participants could also add, redact or comment on their interviews. Not everybody did this so if a transcript was not returned as ‘checked’ (by the participant), they were sent a copy of any relevant outputs and could elect to be named, be anonymised or have the actual texts turned into a descriptive point not attributable to any individual. It was possible too for researchers to ask the participants to develop or add material. This was a very time-consuming process.

In sum, oral history turned out to be insufficient, as there were areas we wanted to be covered in each interview. At the same time, we tried to create a flexible space for participants and how they were represented. To ensure this, we gave participants as much control over their finished transcript as we could. Some made many changes and some made none at all. Nevertheless, there were without doubt aspects that we missed. As already mentioned, we decided that we would use a ‘coding frame’ that would be partly drawn from the theoretical work engaged in and partly with familiarity with a sub-set of texts. The software we used was NVivo 12 and we had two days of bespoke training on this. Our ‘master codes’ were as follows: alternative approaches; collectives; change over time; culture and norms; identity; knowledge; personal journey; power, settings; theorising; mainstream research; user-led research. Each master code had sub-codes to enable us to record particular examples or particular ways of talking about a topic. 

The development of the coding frame was conducted by meeting at least twice a week for 3 months. It was painstaking. Each code was defined and the discussions were minuted. This meant that, if we were having difficulties with codes or chunks of text, we could revisit what we had done before in similar circumstances in the minutes. The length of time this took was partially for certainty but building the coding frame was not a technical exercise only; it involved complex discussions of conceptual matters as well. Once again, this emphasises the inextricable relations between theory and method.

NVivo allows for more than one code to be allocated to a text which means the analysis can be richer. However, it also has a feature called ‘annotations’ which is a space for reflection, suggesting relevant literature and making comparisons between and within transcripts and incorporating relevant literature. Once we had some provisional notion of the importance of a set of codes, this was developed not just through further coding but by making fuller use of the Annotation function.

Coding is often called ‘mechanistic’ so in the next section we will show this is not the case in our analysis of the emergence of mad knowledge.

### Critical Discourse Analysis

I will conclude by returning to the issue that we raised at the start of this paper, concerning the interweaving of theory and methods. Part of our theoretical orientation was critical discourse analysis (CDA). For some theorists CDA is a form of critical sociology. This takes an interdisciplinary approach to the analysis of societal injustices and oppressions. As pointed out earlier, this approach developed in the 1930s with a group of philosophers, cultural analysts and social scientists. It was Marxist in orientation, normative in its values, and sought social change or transformation and empowerment of the dispossessed. Some aspects of this remain valid but, as we pointed out earlier, it was criticized both in the “discursive turn’ in social theory, and in the emergence of smaller groups who considered that their oppressions were distinct and could not be ‘read off’ a general Marxist analysis. Feminists, post-colonial activists and thinkers and disability activists argued in their different ways for the specificities of their subjugation and routes out of it, including the development of counter-narratives. This confluence of the emergence of specific oppressions and activisms and the ‘turn to language’ changed critical theory, such that attention was accorded to the way discourse and other forms of representation reproduced fundamental structures of disadvantage and privilege. Discourse, in this mode of argument, is not just speech but is a systematic way of representing the world or parts of it as embedded in practice. The incorporation of discourse analyses into critical theory began in the 1970s, notably in the work of Fairclough and Wodak ( Fairclough *et al*., 2011; Wodak, 2001). We sought to distance ourselves from the generalist or universalist approach of The Frankfurt School, because CDA is not just a ‘technique’. Thus, as described earlier, we drew on concepts such as situated knowledges ( Haraway, 1988) or ethnocultural niches ( Nazarea, 1999) and hence located ourselves in contemporary critical theory that is concerned with the knowledge and the practices of marginalised groups as understood and developed by them. Consequently, we emphasize that those who are denied a voice should have a seat at the table and speak for themselves and their organizations. 

Today, those closer to the original Frankfurt School privilege the critical theory aspect of the approach whereas those whose roots are in linguistics emphasise discourse. For those coming from the ‘linguistic’ or sociolinguistic background rather than the ‘critical theory’ end, CDA functions more as a toolbox and is not a single and coherent approach ( Gavriely-Nuri, 2017). There is, then, latitude in how CDA is conceptualised and functions, yet it remains the case that CDA takes a social justice approach and that the analysis of power is central ( Mulderrig, 2008). Empowerment is built into the methodology. For us, CDA was useful because it has a base in critical theory but also because it enabled us to draw on concepts specific to madness and to emphasize the intersectionality that our transcripts showed early was an antidote to universalism ( Crenshaw, 1991; Nash, 2008). However, the ‘subjects’ of this research are not accorded any control or power themselves.

To understand how we approached the transcripts and the literature, all three components of CDA are necessary. CRITICAL because we drew on, though modified, the approaches taken in Critical Theory more generally. DISCOURSE, at a simple level because our material was mostly texts. Discourse analysis is a distinct form of working with texts and the term is used broadly. It concerns itself with key semiotic structures in a text, the social ‘grammar’. It is a social product and can define and redefine the meanings that it identifies in how the dominant culture (institutions, boundaries, enactment)) reproduces itself through language and this can be quite superficial or attentive to deep structures and paralanguage. It is centred on language but not usually in the full social sense of a layered embedding in practice. So, we may take a particular text, or set of texts to analyse but, in contradistinction to usual approaches, the ‘background’ work for interviews referred to above means attention to the setting where the discourse is placed is primary and power relations are central ( Mayr, 2008). This is why I have argued that theory and method cannot be separated and certainly do not require distinct methods.

Many tributaries produced the kinds of work now called CDA and they are tributaries of subjugation as well as empowerment. In fact, there is much more literature on the *re*production of cultural structures than there is on new narratives. In the landscape as a whole, the attention to language itself runs from very superficial to intensely detailed. It could be said that our approach was constructivist but we gave a much larger role to the materiality in which language is set than most ( Shankar & Cavanaugh, 2012). And to call an analysis CDA is to look at this embedding. Importantly, CDA is not an analysis of conversations, or dialogues, it is not conversation analysis (CA) which usually aims primarily to show how language acts as a social glue ( Silverman, 2015). Power, if it appears, is interpersonal not structural. Even the body of work which calls itself Foucauldian discourse analysis, while it highlights power relations, still does so at the interpersonal level of conversations ( Arribas-Ayllon & Walkerdine, 2008). But Foucault was not talking about talk! Like other critical work EURIKHA was centrally about change, and took the view that discourse does not just reflect change, it is part of the strategy for bringing it about or preventing it. While CDA aims to empower participants as individuals and groups, this presents a fundamental challenge in relation to those who, on grounds of 'mental illness', are considered deficient not just in their capacity to meet their social obligations, but also in their capacity for rational thought itself. To be excluded from the domain of legitimate knowledge is to concede the battle for truth to those deemed reasonable. To be deemed 'at fault' in one's capacity to know renders the goal of social justice and equality unattainable. One way to start redressing these power imbalances, then, is to recognize that those deemed mad can nonetheless be 'knowledge makers'.

Finally, this is an ANALYSIS. Typically work that uses, for example, thematic analysis will speak of ‘themes emerging’ from the data’ ( Braun & Clarke, 2006). This is an untenable position because the analytic work is always informed, explicitly or not, by theoretical and epistemological underpinnings. To be explicit about calling our approach CDA means it approached the data with some pre-formed social categories that were central to the goal of the project. This is anathema to many qualitative researchers who assert that it is possible to fully analyse a text atheoretically, a position epitomised in grounded theory ( Charmaz, 2014). Such a position denies that such analyses are always *interpretations*, researchers do not come to their material with a completely blank mind and let the data fill it in some unmediated way. There is a huge sociological literature on this issue of ‘bracketing out’ associated with ethnomethodology and now ‘post-qualitative inquiry ( Gerrard *et al*., 2017). The more appropriate strategy, especially with a new field, is to be quite explicit about the framing with which we approach the data. A ‘coding frame’, such as the one described above, is not just a set of categories and sub-categories. It is dynamic and its major categories are theoretically driven, shaped in our case by the theory we have been developing and vice versa. Like any qualitative analysis, these frames need adjustment as analysis proceeds. ‘Empty’ codes are important as I have said. They tell us that certain threads either were not important, or we defined them incorrectly which could lead to misleading interpretations. So, the analysis is informed by critical theory and looks to discourse not as a ‘representation’ of what concerns us but as dynamic, explanatory and generative. This can be at a very abstract level but can also be seen at a micro-level when talk exemplifies the knowledge we are interested in, or indeed, contradicts the original framing. In sum, coding is often called a ‘mechanistic’ approach to qualitative research. We hope to have shown that conceptual and interpretive work is central to the functioning of the analysis.

This recognition must extend to the presentation of the ‘results’. Qualitative research generally involves argument at some level ‘illustrated’ by quotations. This means that meaning units are extracted from the overall transcripts. And some transcripts did lend themselves to this, being highly structured in the way people answered questions and in how they connected these together. Conversely, some transcripts were more a free-flowing narrative and to ‘extract’ a unit of meaning from these means we lose another piece of ‘context’ – that of the interview itself. In future papers, we will take the nature of transcripts into account when comparing them and entering them into *our* interpretation, sometimes summarising what went before but also with the result that some ‘extracts’ are much longer than others. In sum, we believe it is important not to attempt to homogenise at any level but to draw attention to the patterns that result.

## Conclusions

In this paper I have introduced the thinking in EURIKHA around the knowledge produced by people subjugated on grounds of their epistemic competence, subjugated individually, ethically and as a group. I hope that the paper introduces some important features of an approach that might be used to really give voice to the ‘lived experience’ of mental health service users. It also offers sufficient detail to orient the reader to the ways that the more substantive papers to come have a solid base in critical thinking and critical practice. The aim is to show that new knowledge is being generated from the kinds of sources explored in this paper and are part of the drive to change the variable conditions of existence of people who are ‘othered’ because they do not meet the criteria of the logical, social or moral attributes seen as necessary for society to function. To develop counter-narratives is to unsettle and trouble the privilege given to some ‘experts’ which can be harmful in the overall life of a person or persons. In that sense EURIKHA seeks to facilitate a new imagining of those of us designated mad.

### Coda

As science and technology studies has definitively shown, when a project or programme of research is made public, many things are left unsaid or glossed over. The descriptions in this paper are no exception. Many issues of power and knowledge gave rise to what I have written here but also what we have not written. To write a reflexive piece on the social and political, ethnic and gender positionalities in the team would entail making public very troubled issues and interactions. This is not unique to this project and again speaks to the power of silence. But It will be seen as no accident that the author on this paper, who was also the PI on the project, ultimately took the decision to configure the dissemination of the work. There is more to come including an examination of the counter-narratives that are being sustained despite attempts to co-opt them into mainstream research and thought. It will be up to the readers to decide if EURIKHA's aspiration to amplify this counter voice has, at least partially, succeeded.

## Data availability

The data is not publicly available because of legitimate concerns about confidentiality expressed by some participants as a condition for their participation. While some participants wanted to be named, others were extremely worried that they would be identified and did not want to be named. Given the difficulties this presented for anonymisation, I took the decision to use full anonymisation. As participants could be identified from the transcripts of their interviews, this also meant that the transcripts could not be made available and these are the only form of data collected for this study. No view on data sharing was expressed by the Ethics Review Board. Any properly qualified researcher who wishes to access some of the data for legitimate reasons should contact the author of this paper individually to discuss access and conditions ( Diana.Rose@anu.edu.au).
