# Supplementary material for: Critical qualitative research on ‘madness’: knowledge making and activism among those designated ‘mad’
Source: Wellcome Open Res. 2021 May 6;6:98. [Version 1] doi: 10.12688/wellcomeopenres.16711.1 (PMC8314133; doi:10.12688/wellcomeopenres.16711.1)
Supplement: Peer review history

# Peer review: Reviewer response for version 1

**DOI:** 10.21956/wellcomeopenres.18428.r44955

**Published:** 2021-07-26

**Authors:** Mark Cresswell

**Affiliations:** 1 Bath Spa University, Bath, UK

Diana Rose’s Open Letter in the Wellcome Open Research series outlines a completed research project (‘EURIKHA’) of great significance for critical studies of mental health. EURIKHA interviewed activists who have been exploring alternative forms of knowledge of ‘madness’ beyond conventional psychiatric paradigms. Rose’s outline focuses on that part of the project which dealt with the Global North including 48 interviews with activists in North America, England, Australia, New Zealand, and Europe. Outputs resulting from this research are eagerly anticipated.

 The editorial team of this review process have asked me to focus upon five questions in response to Rose’s letter:

1. Is the rationale for the Open letter provided in sufficient detail?
2. Does the article adequately reference differing views and opinions?
3. Are all factual statements correct and are statements and arguments made adequately supported by citations?
4. Is the Open Letter written in accessible language?
5. Where applicable, are recommendations and next steps explained clearly for others to follow.

My answers to 1 and 4 are an unqualified ‘yes’: a full and lucid account of the research has been provided including reference to many fascinating issues and dilemmas. Rose provides thoughtful guidance about how to reflexively approach a difficult and under researched area and this will benefit any researcher investigating ‘lived experience’ and political activism as well as mental health. These issues require no further comment. I also do not intend to respond to the many methodological questions that the project raised. Although Rose writes at length about methodology, I thought that the combination of purposive sampling and a modified oral history approach involving the ‘coding’ strategies of qualitative research was persuasive. So, in the rest of this review I will concentrate on questions 2 and 3, about which I have some critical comments to make. These concern the definition of ‘knowledge’, the role of ‘practice’, the relation of universities to social movements, some remarks on Foucault and, finally, cultural change within psychiatry in the period which the project’s interviews cover (that is, since 1970). I have not answered question 5 as the project is completed and it is for the EURIKHA team to decide the next steps.

**Knowledge(s)**

****

 This was the central theoretical category of the project’s research. Yet it seems under-developed in Rose’s outline. Key components in a theory of social knowledge - that it labels and classifies human experience in the context of a significant claim to truth - are under-emphasised. The metaphor of surface and depth is employed, instead, to illuminate the core goal of the project: ‘to map the knowledge being generated across the world by people silenced for centuries’, with ‘surface’ being used in its verb rather than its noun form (‘our task was to surface the positive knowledge of survivors’ [ibid: 4]). But this runs the risk of undermining what Rose otherwise insists on: the socially constructed condition of knowledge. The weakness of the metaphor of surface and depth - together with the narrative of ‘silence’ and ‘voice’ which accompanies it - is that it sets up a false dichotomy. On the one hand, we have a socially constructed psychiatry represented by its textual ‘bible, the *Diagnostic and Statistical Manual of Mental Disorders *(DSMs) (for example, American Psychiatric Association [APA], 2013 ^1^); but, on the other hand, there is a newly liberated ‘mad’ knowledge of indeterminate content. Surprisingly, given the aspiration to unleash ‘positive knowledge’, Rose defines this more in terms of its conditions of subjugation than its actual content; thus, ‘mad people are imagined as lacking rationality...tacit assumptions about lack of capacity’ etc. (ibid: 1 & 3). Later, discussing the theoretical framework of critical discourse analysis (CDA), Rose admits that the research team approached the interview data with ‘pre-formed social categories’ (ibid: 11) but this only concedes what could hardly be denied with the employment of *any* interpretative framework - that the researchers were analysing the data partly in terms of the theory. This, though, leaves undescribed the actual conditions of possibility of survivor-knowledge, except we are informed that it has now ‘surfaced’.

 It could be objected here that the ‘positive knowledge of survivors’ will be fully described as ‘situated knowledge’ in future EURIKHA outputs and not just referred to generically, as it is here, as having ‘form’ and ‘content’ with ‘symbolic and material requirements’ (ibid: 4). But it would have been useful to have seen some practical examples. Without that, even in outline, the danger is that the tendency to ‘valorise’ survivor-knowledge overrides the need to analyse its emergence with as much acuity as we readily apply to the DSMs. Both survivor-knowledge and the DSMs are socially constructed. This is just another way of insisting that the two forms of knowledge are epistemologically equal and should be treated as such.

**Practice(s)**

****

 Rose takes it as a given that both the subjugating (psychiatry) and the subjugated (the ‘mad’) phenomena are forms of knowledge and therefore that the central issue at stake is knowledge(s). Is that true? It has to be partially true insofar as ‘activist...groups had provided analyses of experiences that reconfigured...venerated psychiatric diagnosis’ (ibid: 5) and Rose rightly references the activism of Pembroke and the re-articulation of ‘ *deliberate* self-harm’ as just ‘self-harm’ in the late 1980s/early 1990s. But interpreting this as a contestation just at the level of knowledge misapprehends lived experience. Pembroke was concerned with a set of abusive practices - for example, the mistreatment of individuals who self-harm in emergency departments in England - and their replacement by an alternative set of practices (reforming attitudes, pioneering ‘harm-minimisation’ approaches etc.). There seems to be confusion about the temporality of the process of experience>knowledge>practice: Rose posits an experience which becomes knowledge and is then enacted as a practice’ (for example, ‘self-help groups’ [ibid: 5]). However, it is unlikely that the relation of experience/knowledge/practice is linear: if an experience comes first then it is certainly possible that an alternative practice may predate the articulation of knowledge which then creates the conditions of possibility for a new experience etc. The relation of knowledge to practice in the sequence experience>knowledge>practice is empirical: it may only be determined through specific historical inquiries, and it is to be hoped that forthcoming EURIKHA outputs shed new light on this process. This reviewer is not convinced that the centrality of the concept of ‘knowledge’ facilitates that. Rather, a focus upon social practice that embodies knowledge rather than articulating it, may help break the dichotomy between knowledge and practice. Such a concept is found, for example, in the ‘habitus’ of Bourdieu’s (1980) ^2^ sociology and has been elaborated for mental health social movements in the UK in the academic work of Crossley (2004) ^3^ and his notion of ‘oppositional habitus’. I am not suggesting here that EURIKHA took a wrong theoretical path – just that the emphasis on knowledge needs to be tempered by an equal attention to practice(s).

****

**Universities and Social Movements**

 Mention of Crossley’s work on mental health social movements raises the question of the relation of EURIKHA to the academy. Rose notes this issue but underplays its complexity. The ‘positionality’ of the EURIKHA researchers does seem unique: activists with lived experience of mental distress researching the knowledge-production of activists within mental health social movements whilst located in an elite university which Rose characterises as, ‘the leading psychiatric research institution in Europe’ (Rose, 2021: 7). Almost a decade ago, when Spandler and I (Cresswell and Spandler, 2013 ^4^) considered similar issues, we thought that such a position entailed what we called ‘lived contradictions.’ The researcher was simultaneously: 1) treating their research subjects as agents of change but objects of knowledge; 2) expressing solidarity with those they researched whilst employing certain ‘outputs’ to increase academic prestige; 3) respecting the knowledge of activists whilst yet imposing a theoretical framework upon them. Confronting such contradictions, we argued, involves academics who are engaged with the social movements they study entering into complex relations with those movements. But as we pointed out, specifically referencing Rose’s work, ‘the survivor academic who is doubly located’ - within the university but also within the movement - ‘faces a number of highly specific ‘lived contradictions’ which we do not address here, but which deserve to be considered in their own right’ (Cresswell and Spandler 2013: 152 ^4^). We did not address them because we did not have the experience to do so. Perhaps it could be the case that the conclusion of the EURIKHA project provides an occasion to address some of these issues by academic-activists who do have direct experience of these contradictions?

**Foucault**

 I have two brief remarks on Foucault. The first is scholarly. I agree that it remains important to keep using his work as a launchpad for inquiry in mental health studies. Where this is the case, we now have the full unabridged translation of his *History of Madness *(2013) ^5^ and I think this should be the sourcebook used rather than the earlier, abridged translation, *Madness and Civilization* (1967).

 The second remark concerns the limitations of the narrative of ‘silence’ and ‘voice’. Rose employs *History of Madness *to establish the historical silencing of madness by psychiatry. Later, in the first volume of his *History of Sexuality, *Foucault makes a different point about ‘sex’; unlike, madness, which has been silenced, sex has been the object of a ‘discursive explosion’ (Foucault, 1978: 17 ^6^) since at least the 19 ^th^ century. I wonder if we could not say the same about ‘mental health’ today. It is spoken of everywhere – in higher education, sport, social media, for example – so that cultural change within the mental health system has been notable in the time period covered by EURIKHA’s interviews. It may be the case, then, that the narrative of ‘silence’ and ‘voice’ needs to be supplemented by a focus upon a cultural milieu in which mental health-talk is ubiquitous. One example would be the anti-stigma campaign Time for Change; ostensibly, this was a progressive intervention, but it has been critiqued as tacitly reinforcing the epistemology of the DSMs because the notion of ‘mental disorder’ that underpins its activism goes unchallenged (see McWade, 2019 ^7^). The issue here is not about ‘silencing’ but, rather, permitting the expression of ‘voice’ only within constrained limits. This brings me on to a final point about cultural change within psychiatry and society within the timeframe that the EURIKHA project covers.

**Cultural change and psychiatry**

 EURIKHA’s objectives are wide in scope including the aspiration ‘to compare the knowledge produced by service users to mainstream knowledge about ‘mental illness’’ (Rose, 2021: 3). This assumes an awareness of mainstream knowledge – but Rose provides little indication that EURIKHA was up to date with this. Their outline still refers to ‘mental illness’ which was not even in operational use as far back as DSM-I in 1952 (APA, 1952 ^8^). In fact, the mental health system has undergone profound change in the period covered by EURIKHA including: 1) the move from an asylum regime to one of community care; 2) the expansion of the DSMs particularly in terms of the ‘common mental disorders’ (anxiety and depression); 3) a pharmacological revolution especially in terms of the prescription of anti-depressants and anxiolytics; 4) an explosion of media representations of distress especially since the advent of social media; 5) political transformations including the emergence of survivor-led social movements which is one of EURIKHA’s main themes. Not all of these transformations have taken place within psychiatry; many, such as the explosion of social media representations of mental distress, refer to wider cultural change. Without referring to these transformations it is unclear how EURIKHA could compare mainstream and survivor knowledge. Whether they can meet that objective remains to be seen.

 My final point concerns the heterogeneity of service users within the mental health system, which I think Rose under-emphasises. In terms of England, nearly 3 million people were in contact with secondary mental health services in 2019-20 ( NHS Digital, 2021). This number subsumes a wide range of diagnoses and experiences, ranging from the provision of psychotherapy to detention under the Mental Health Act (1983). Given this heterogeneity, I still feel a little unclear about who EURIKHA actually interviewed. They do not discuss it in the way that mainstream qualitative research establishes ‘inclusion’ and ‘exclusion’ criteria. Rose (2021: 4) does clearly state, disparagingly, in discussing the ‘stories’ of service users experiences that ‘only certain ones will do – mostly ones of recovery made possible by a psychiatrist or medication.’ I assume from this that their 48 interviewees had experienced psychiatry negatively, been harmed by it, and were part of the user/survivor/mad movements. But Rose never actually says so directly and it would have been useful for them to do so. It would have oriented the reader more specifically and prepared them for the outputs to come.

 Despite these criticisms, EURIKHA remains a landmark study and its future outputs are keenly awaited. Ironically, given the criticism of universities outlined above and echoed in some of my work, it is unlikely that such a wide-ranging, resource-intensive project could have been brought to fruition without the involvement of a mainstream funder and an elite university. EURIKHA’s ultimate goal, ‘to lay out…the lineaments of survivor knowledge in the early 21 ^st^ century’ is one many survivors and academics will be eager to see achieved.

Does the article adequately reference differing views and opinions?

Partly

Are all factual statements correct, and are statements and arguments made adequately supported by citations?

Partly

Is the Open Letter written in accessible language?

Yes

Where applicable, are recommendations and next steps explained clearly for others to follow?

Not applicable

Is the rationale for the Open Letter provided in sufficient detail?

Yes

Reviewer Expertise:

Sociology

I confirm that I have read this submission and believe that I have an appropriate level of expertise to confirm that it is of an acceptable scientific standard.

## References

[1] : Diagnostic and Statistical Manual of Mental Disorders.2013; 10.1176/appi.books.9780890425596 10.1176/appi.books.9780890425596

[2] : The Logic of Practice. *Stanford University Press* .1980;

[3] : Not being mentally ill. *Anthropol Med* .2004;11(2) : 10.1080/13648470410001678668 161-80 10.1080/13648470410001678668 26868200

[4] : The Engaged Academic: Academic Intellectuals and the Psychiatric Survivor Movement. *Social Movement Studies* .2013;12(2) : 10.1080/14742837.2012.696821 138-154 10.1080/14742837.2012.696821

[5] : History of Madness.2013; 10.4324/9780203642603 10.4324/9780203642603

[6] : The History of Sexuality: Volume 1. *New York: Pantheon Books* .1978;

[7] 10. Madness, Violence, and Media.2019; 10.3138/9781442629981-015 150-164 10.3138/9781442629981-015

[8] : Diagnostic and Statistical Manual of Mental Disorders.1952;

# Peer review: Reviewer response for version 1

**DOI:** 10.21956/wellcomeopenres.18428.r43764

**Published:** 2021-06-04

**Authors:** Junko Kitanaka

**Affiliations:** 1 Department of Human Sciences, Faculty of Letters, Keio University, Tokyo, Japan

Professor Rose’s paper is a very welcome addition and significant contribution to our understanding of the current state of user-led research. It sketches out, step-by-step, the actual processes of how to perform user-led research from the viewpoint of a veteran in the field. It clarifies the theoretical stance of the “Mad Studies”, destabilizes some of the assumptions implicit in academic research, and critically investigates the power differentiation between the academics and non-academics in the politics of scientific knowledge production. The paper reads as a testimony to the many challenges one must contemplate before embarking on this kind of research. It is thus a guiding light, particularly for scholars outside the immediate circles of user-led research in the UK, US, and Canada, including those who attempt user-led research in the so-called Global South.

 Thus, while it is certainly an important paper that accomplishes what it sets out to do, it is also rather curious in that it neglects to incorporate or highlight much of the significant progress in the field over the last few decades. Instead, Professor Rose begins her theoretical section by drawing on Foucault and the Frankfurt School to argue that the voices of the "mad" have been historically silenced and are still silenced today. While that may still be true to an extent, much has changed since the time of Foucault, and this is in large part thanks to the work of people like Professor Rose and her colleagues. The curious lack of recognition of this progress seems problematic not only because it paints a lopsided view of Mad Studies (the 'mad' have been listened to, but her paper does not give that impression), but also because it likely leaves other researchers wondering what to think about this progress: what they have overcome, what issues still remain, and how they are dealt with in the current EURIKHA project. 

 There are three areas of progress in particular that I’d like to point out with regard to the famous ECT research done by Professor Rose *et al.*, published in BMJ ^1^. First of all, this work was epoch-making in the sense that it destabilized the politics of evidence. It problematized how the narratives (voices) of the people with mental illness had been treated as mere “anecdotes” and placed at the bottom of the hierarchy in the order of scientific knowledge. Their voices were suppressed and/or marginalized partly because they were seen as irrational, emotional, and too subjective. Secondly, the ECT research demonstrated how science, by neglecting users’ subjective voices, produced quasi-scientific knowledge that simply failed to explain and/or understand users’ lived experiences. By closely investigating how science failed—for instance, by asking patients about their “satisfaction” when they were feeling most vulnerable and pressured to answer positively (such as right after they were given ECT)—Professor Rose *et al*. ^1^ showed why some scientific findings might be at odds with users’ lived experiences. Third, the ECT research offered innovative methodologies by showing how to collect users’ own voices as a bundle and turn them into a form of evidence, a sign of scientific objectivity. In other words, the ECT research democratized scientific research by showing how the “mad” can be the agents of scientific knowledge production.

 Of course, some of these insights are reflected in Professor Rose’s paper on EURIKHA. For instance, the paper raises the fundamental questions of what is good science and whom science is for. It elaborates on the continued elitism in science, deconstructs the process of ethics-review where such power dynamics are inscribed, and points to the sensitive uses of terminology. Yet, instead of a celebratory tone, what one gets from this paper is an overwhelming sense of the painstakingly difficult labor involved in this kind of research. It conveys the message that the attempts to fully listen to diverse opinions, to try to include as many voices as possible, to attend to the complexities can be a truly daunting task. The resulting levels of emotional woundedness seem like they could thoroughly exhaust the researcher. And perhaps, the most important and delicate question remains unanswered: what to do with a certain sense of sacredness that comes with users’ lived experience, their vulnerability, and trauma?

 I wonder if Professor Rose’s ambivalence and a certain silence around these issues may in fact be a product of progress in the field. That is, the progress made from the time when it was enough to criticize science and medicine, to the current time when that critical gaze is turned back on social scientists themselves. Social scientists, just as their natural scientist cousins, also tend to prioritize reason, avoid bare emotion and passion, and/or mold them into scientifically appropriate forms. As David Mosse (2018) ^2^ has pointed out with regard to PPI, what gives people’s illness narratives the power they have—their subjective, specific aspects—easily gets lost when reformulated and presented in a scientific manner. Further decontexutalized as data, such narratives may also lose some of their appeal, ceasing to be the kind of knowledge that could have the power to transcend science.

 Another tension that comes from scientificizing user-led research may also be the politics around positionality and power dynamics. More specifically, what to do with the all-too-powerful view of a scientific expert who also speaks for and as the victim. At least in Japan, the context that I am most familiar with, there is an increasing presence of expert-users, doctors who have themselves experienced a psychiatric illness (such as developmental disorders, depression and dementia) and have come out as scientist/clinician/users. While they are highly praised for their courage, and appreciated for their unique expertise, they sometimes seem to evoke ambivalence among other users because these expert-users’ voices may carry almost too much power and moral weight. Some may see that, merely by the fact that they have obtained M.D. and/or Ph.D., they do not really know what it is like to live with an illness and stigma. Their elite position may even be seen as a sign of arrogance that disqualifies them as true messengers in the first place. Particularly in the era of SNS, when some people may no longer need an “expert” to represent them, when the figure of the “patient” has changed radically from that of a passive silenced existence to an active agent who can access medical knowledge, what do such divisions mean and how have they changed the dynamics of user-led research and/or the possibilities for scientific knowledge production?

 Lastly, I wonder if Professor Rose could elaborate a bit more about both the divide and also bridges between the Global North and the Global South. It is unfortunate that results from the so-called Global South were not incorporated in this report, particularly because the effects of previous user-led research are already apparent in some areas (Sugiura et al. 2020) ^3^. There are also other unique attempts of user-led work called tojisha kenkyu (self-support research) that have emerged locally in Japan (Ishihara 2015) ^4^, where practitioners are beginning to compare their work to what has been done in the UK. In Japan, for instance, people with dementia are increasingly speaking up, writing monographs, using SNSs, and their opinions are actively sought after by the government and corporations seeking to create universal designs and dementia-friendly cities in a super-aging society (Kitanaka 2020) ^5^. The same can be said for people with developmental disorders, whose growing presence in schools and workplaces has challenged the way people think about mental health in general (Kumagaya 2016) ^6^. People with schizophrenia in Hokkaido, at a place called Bethel, have surprised the public by performing “hallucination/delusion contests” (where the person with the most hilarious and/or moving delusional experience gets the first prize) and have turned the act of talking about illnesses into public performance, a source of empowerment, and a space of mutual laughter and shared experience (Nakamura 2013) ^7^. Some of these people have since sought ways to turn their knowledge into scientific evidence. People like Shinichiro Kumagaya, one of the leaders in the tojisha movement, are also expanding their insights about their vulnerabilities into projects for the “majority” to study themselves, creating personnel-developing projects for universities and corporations. Especially during the COVID-19 pandemic, they have begun to effectively challenge “mainstream” people to imagine what it is like to live with disabilities, and to ask themselves what we need to do when everyone is found to be vulnerable in some ways.

 Given these new developments, I hope that Professor Rose might consider, in this EURIKHA report, including at greater length recent advances that have been made, assess where they have succeeded in changing the way scientific knowledge is produced, and what issues we still face in creating future directions for user-led research.

Does the article adequately reference differing views and opinions?

Yes

Are all factual statements correct, and are statements and arguments made adequately supported by citations?

Yes

Is the Open Letter written in accessible language?

Yes

Where applicable, are recommendations and next steps explained clearly for others to follow?

Partly

Is the rationale for the Open Letter provided in sufficient detail?

Yes

Reviewer Expertise:

medical anthropology

I confirm that I have read this submission and believe that I have an appropriate level of expertise to confirm that it is of an acceptable scientific standard.

## References

[1] : Patients' perspectives on electroconvulsive therapy: systematic review. *BMJ* .2003;326(7403) : 10.1136/bmj.326.7403.1363 1363 10.1136/bmj.326.7403.1363 12816822PMC162130

[2] : Can the Experience of Participatory Development Help Think Critically about ‘Patient and Public Involvement’ in UK Healthcare?. *Sociological Research Online* .2019;24(3) : 10.1177/1360780418797718 444-461 10.1177/1360780418797718

[3] : An end to coercion: rights and decision-making in mental health care. *Bull World Health Organ* .2020;98(1) : 10.2471/BLT.19.234906 52-58 10.2471/BLT.19.234906 31902962PMC6933425

[4] : Learning from tojisha kenkyu: Mental health “patients” studying their difficulties with their peers. *T. Shakespeare (Ed.), Disability Research Today International Perspectives* .2015;27-42

[5] : In the Mind of Dementia: Neurobiological Empathy, Incommensurability, and the Dementia Tojisha Movement in Japan. *Med Anthropol Q* .34(1) : 10.1111/maq.12544 119-135 10.1111/maq.12544 32311787

[6] : Individual impairment vs. disability that depends on location and era: Research on autism spectrum disorder. *Japanese Journal of Developmental Psychology* .2016;27(4) :322-334

[7] : A Disability of the Soul: An Ethnography of Schizophrenia and Mental Illness in Contemporary Japan. *Ithaca: Cornell University Press* .2013;
